# Associations in intentions to use anabolic-androgenic steroids among non-consuming boys and men with probable eating disorders and muscle dysmorphia

**DOI:** 10.1186/s40337-025-01435-3

**Published:** 2025-11-11

**Authors:** Kyle T. Ganson, Timothy Piatkowski, Alexander Testa, Jason M. Nagata

**Affiliations:** 1 Factor-Inwentash Faculty of Social Work, University of Toronto, Toronto, ON Canada; 2 School of Applied Psychology and Griffith Centre for Mental Health, Griffith University, Brisbane, Australia; 3 Centre for Health Services Research, The University of Queensland, Brisbane, Australia; 4 Department of Management, Policy and Community Health, University of Texas Health Science Center at Houston, Houston, TX USA; 5 Department of Pediatrics, University of California, San Francisco, San Francisco, CA USA

**Keywords:** Anabolic-androgenic steroids, Eating disorders, Muscle dysmorphia, Boys, Men

## Abstract

**Background:**

Anabolic-androgenic steroids (AAS) are used to achieve a muscular and lean body to align with current ideals for boys and men. Identifying associations with intentions to use AAS, a precursor to actual use, is critical for prevention efforts. This study aimed to examine whether boys and men with a probable eating disorder or probable muscle dysmorphia have stronger intentions to use AAS, and whether these intentions differ between these conditions.

**Methods:**

Data from a sample of 1,515 participants from the Study of Boys and Men who had never used AAS were analyzed. To address the study aims, one adjusted linear regression analysis was conducted along with post-hoc Wald tests.

**Results:**

Probable anorexia nervosa/atypical anorexia nervosa, bulimia nervosa, and muscle dysmorphia were associated with greater intentions to use AAS compared to those with none of these conditions. Post-hoc Wald tests revealed that there were no significant differences in intentions to use AAS between those with probable anorexia nervosa/atypical anorexia nervosa, bulimia nervosa, and muscle dysmorphia; however, there were significant differences between these conditions and probable binge-eating disorder.

**Conclusion:**

The findings from this study add to a growing literature underscoring similar muscularity-oriented features across anorexia nervosa/atypical anorexia nervosa, bulimia nervosa, and muscle dysmorphia. Assessment of intentions to use AAS may be warranted among boys and men with eating disorders and muscle dysmorphia to ensure the provision of prevention and early-intervention strategies.

**Supplementary Information:**

The online version contains supplementary material available at 10.1186/s40337-025-01435-3.

## Abstract (plain language summary)

This study explored whether boys and men who have a probable eating disorder or muscle dysmorphia are more likely to intend to use anabolic-androgenic steroids (AAS) than those without. Data from over 1,500 participants who had never used AAS before were analyzed. The results showed that those with probable anorexia nervosa/atypical anorexia nervosa, bulimia nervosa, or muscle dysmorphia were associated with greater intentions to use AAS compared to those without these conditions. Interestingly, participants with probable binge-eating disorder did not exhibit significant intentions to use AAS. These findings suggest that boys and men with certain eating and body image concerns may be especially vulnerable to AAS use. Identifying these individuals early could assist healthcare professionals in providing the appropriate support and prevention strategies.

**Supplementary Information:**

The online version contains supplementary material available at 10.1186/s40337-025-01435-3.

## Introduction

Anabolic-androgenic steroids (AAS) are commonly used without medical supervision to enhance muscle growth [1, 2]. Although illegal to produce or sell in Canada and the United States (U.S.), lifetime use remains relatively high, particularly among boys and men, with estimates ranging from 1.4% to 6.7% [3–6]. The use of AAS can have numerous detrimental biological, psychological, and social effects. For example, AAS use is associated with cardiovascular and neuroendocrine issues, mood disorders, suicidal behavior, and dependence symptoms, as well as criminal offending, aggression, and sexual risk behaviors [2, 7–11]. Understanding the factors associated with intentions to use AAS, which is a precursor to actual use, is critical to inform prevention efforts.

One worthy area of investigation is the associations between eating disorders (ED), such as anorexia nervosa (AN), bulimia nervosa (BN), and binge-eating disorder (BED), and muscle dysmorphia (MD), which is a specifier of body dysmorphic disorder, and intentions to use AAS. Both EDs and MD are most common among adolescents and young adults, with a mean age of onset in young adulthood (i.e., 19–21 years) [12, 13]. The prevalence of EDs and MD among boys and men widely ranges depending on the sample composition and measurement of these conditions [14]. However, it has been estimated that 12.8% of boys in Australia [15] and 21.3% of boys and men in Canada and the U.S. experience any ED, while 2.2% of boys in Australia [16], 2.8% of boys and men in Canada and the U.S [17]. , and 1.3% of undergraduate men in Spain [18] experience MD.

While EDs and MD are distinct conditions, a recent meta-analysis documented that MD is positively associated with ED symptoms [19], and there have been calls for the reclassification of MD to align more closely with EDs [20]. The overlapping features of MD and EDs, particularly with AN and BN, are primarily driven by underlying body image concerns, body checking, and the engagement in behaviors to change one’s body to align with the sociocultural ideal [21, 22]. Indeed, for boys and men with both an ED and MD, body image concerns primarily focus on muscle dissatisfaction and drive for muscularity [23–27]. Therefore, boys and men with these conditions may engage in muscularity-oriented behaviors, such as excessive weight training, dietary changes (e.g., protein overconsumption), and the use of dietary supplements, to alter their body, weight, and shape to be more muscular [28–30].

Given AAS’s documented effectiveness at increasing muscle mass [31], boys and men with an ED or MD may also be motivated to use these drugs to address their muscle dissatisfaction and achieve their body goals. While there is research documenting the positive association between MD and AAS use among boys and men [32–34], as well as some null findings [35, 36], there is little research on whether boys and men with EDs are more likely to use AAS. Some research suggests that AAS use and intentions to use AAS are positively associated with ED psychopathology [37–39]. As a result, it has been posited that AAS use should be considered a component of disorders such as EDs or MD [40].

Ultimately, the intentions of boys and men, with or without an ED or MD, to use AAS may be driven by sociocultural theories, such as the Tripartite Influence Model [41], which underscores that pressures from family, peers, and the media influence the internalization of body ideals, body dissatisfaction, and, therefore, the engagement in risky muscle-building behaviors (i.e., AAS) aimed at changing one’s body. Prior research has successfully applied this model to explain poor body image, disordered eating behaviors, and muscularity-oriented eating behaviors among men [42–45], as well as AAS use among boys [46]. This model has also been expanded to include additional sociocultural influences, such as masculine gender norms [47, 48], which are highly significant when considering male body image, muscularity, and ED and MD symptomatology among boys and men [36, 49–51].

Collectively, existing work highlights the value of sociocultural frameworks in explaining risky muscle-building behaviors. However, the role of EDs and MD in shaping intentions to use AAS remains underexplored, especially in community samples of boys and men. To advance this understanding, the present study aimed to investigate: [1] whether boys and men with an ED or MD have greater intentions to use AAS compared to those with neither condition, and [2] whether AAS use intentions differ between ED and MD groups. For aim 1, we hypothesized that those with an ED or MD would report higher intentions to use AAS compared to those with neither condition. This hypothesis is supported by the notion that those with greater body dissatisfaction and preoccupation (i.e., those with an ED or MD) would be more inclined to use AAS to address these concerns. For aim 2, we hypothesized that those with AN or atypical anorexia nervosa (AAN), BN, and MD would have greater intentions to use AAS than those with BED. This hypothesis is supported by the overall strong body image concerns underpinning AN/AAN, BN, and MD when compared to BED [21, 22, 25].

## Methods

Data from The Study of Boys and Men, an online survey distributed via Qualtrics that aimed to capture data on the body image experiences of boys and men in Canada and the U.S., were analyzed for this study. Data were collected from March 2024 to April 2024 using Instagram and Snapchat advertisements, targeting males between 15 and 35 years old. Advertisements aimed to recruit a broad range of participants (i.e., “Don’t miss your chance to make your voice heard! Participate today in: The Study of Boys and Men, an international research study of the contemporary lives of boys and men.”). Several recommended anti-bot methods (e.g., honeypot questions, attention checks, reCAPTCHA verification) were employed to improve data quality [52]. The initial sample consisted of 1,791 respondents, of which 238 were removed during data validation and screening, resulting in a final dataset of 1,553 participants. This analysis further excluded participants who had reported lifetime use of AAS, resulting in a final analytic sample of 1,515 participants. All respondents provided informed consent via a checkbox. The study was approved by the Health Sciences Research Ethics Board at the University of Toronto (Protocol #45880), which allowed for the recruitment of participants < 18 years and in both Canada and the U.S.

### Measures

#### Intentions to use AAS

Intentions to use AAS were measured based on responses to five items following the prompt, “To what extent do you agree or disagree with the following statements about anabolic-androgenic steroids?” Items included, “I plan to use anabolic-androgenic steroids in the future.” (*M* = 1.53, *SD =* 1.12); “I have looked up information on types of anabolic-androgenic steroids and how to use them.” (*M* = 1.96, *SD =* 1.74); “I have looked up information on how to obtain anabolic-androgenic steroids.” (*M* = 1.51, *SD =* 1.31); “I’ve talked with people who use anabolic steroids about getting or using anabolic-androgenic steroids.” (*M* = 1.52, *SD =* 1.30); and “I’ve learned about getting and using anabolic-androgenic steroids.” (*M* = 1.80, *SD =* 1.60). Response options followed a 7-point Likert scale ranging from *Strongly disagree* [1] to *Strongly agree* [7]. A sum score was created by adding all five items (scores ranging from 5 to 35), with higher scores indicating greater intentions to use AAS. This measurement tool was adapted from prior research that developed it to explore “precursor steps to initiating AAS” among a sample of college men ages 18–29 [53], given the higher prevalence of AAS use among this population [6, 54]. Internal consistency of the sum score using Cronbach’s alpha was α = 0.87. See Supplementary Table 1 for full item details on the intentions to use AAS measure. We also conducted exploratory factor analysis on the five items assessing intentions to use AAS using principal factor extraction. A one-factor solution was specified a priori [53], consistent with initial eigenvalue inspection. The one-factor model produced an eigenvalue of 3.08, accounting for 61.6% of the total variance. All five items loaded strongly on the factor, with standardized loadings ranging from 0.68 to 0.85. Item-level uniqueness values ranged from 0.28 to 0.54, indicating that the factor explained a substantial proportion of the variance in each item. These results support the unidimensionality of the intentions to use AAS construct (see Supplementary Table 2 for factor loadings and uniqueness values).

#### Eating disorders

AN, BN, and BED were operationalized based on an algorithm outlined by Mitchison et al. (2020), which aligned with the *Diagnostic and Statistical Manual of Mental Disorders*, 5th Edition (DSM-5) [21]. Most symptoms, including attitudes and behaviors, were assessed via the Eating Disorder Examination Questionnaire (EDE-Q) 6.0 [55], which has been validated among boys and men across genders, sexual orientations, and ages as young as 15 years [56–59]. The original research team also generated items measuring binge-eating distress and related features (e.g., eating rapidly, eating alone due to embarrassment) [15]. Due to small numbers, AN and AAN were combined. Full operationalization details appear in the open-access publication by Ganson et al. (2025) and in Supplementary Table 3. As no formal diagnostic interviews were conducted, individuals meeting criteria were categorized as having a “probable” ED.

#### Muscle dysmorphia

MD was determined using an algorithm adapted from Mitchison et al. (2021), based on criteria outlined by Pope et al. (1997) and aligned with DSM-5 criteria [21]. The algorithm consisted of validated measures, including the Muscle Dysmorphic Disorder Inventory (MDDI) [60], the Drive for Muscularity Scale (DMS), which was developed with participants as young as 13 years [61], the EDE-Q 6.0 [55], the Pediatric Quality of Life Inventory (for both adolescent and young adult populations) [62, 63], and the Kessler Psychological Distress Scale [64], which has shown strong reliability among adolescent populations [65]. For full operationalization, see the open-access publication by Ganson, Mitchison, et al. (2025) and Supplementary Table 4. As no formal diagnostic interviews were conducted, individuals meeting criteria were categorized as having “probable” MD.

#### Sociodemographic variables

Sociodemographic variables were assessed using multiple self-report items, including age, gender (determined using both sex at birth and current gender identity), race/ethnicity, sexual orientation, and highest completed education. BMI was calculated (kg/m^2^) based on self-reported height and weight. Postal codes (Canada) and zip codes (United States) were also collected to identify country of residence. See Supplementary Table 5 for full details on demographic survey items.

### Statistical analysis

To address aim one, one linear regression analysis was conducted to determine the association between meeting criteria for a probable eating disorder, probable MD, or neither (coded as 0 = None, 1 = AN/AAN, 2 = BN, 3 = BED, 4 = MD) as the independent variable and intentions to use AAS as the dependent, adjusting for the sociodemographic variables. To address aim two, post-hoc Wald Tests were conducted after the linear regression analysis to determine differences in associations between probable EDs and probable MD on intentions to use AAS scores. Statistical significance was set a *p* < .05 and all analyses were conducted in 2025 using StataMP 18.

## Results

Descriptive statistics are displayed in Table 1 (see Supplementary Table 6 for descriptives by age and country). Results from adjusted linear regression analyses showed that those with probable AN/AAN (*B* 3.24, 95% CI 1.05, 5.43, *p* = .004), probable BN (*B* 3.26, 95% CI 1.82, 4.71, *p* < .001), and probable MD (*B* 3.16, 95% CI 1.31, 5.01, *p* = .001), compared to those with none of these conditions, had significantly greater intentions to use AAS after adjusting for relevant sociodemographic variables (Table 2). There was no significant association between probable BED and intentions to use AAS. Effect modification on intentions to use AAS was explored for age, gender, sexual orientation, race/ethnicity, and country, with no significant findings.

**Table 1:** Descriptive characteristics of a sample of boys and men from Canada and the united States who never used steroids (*N* = 1,515)

	Overall %
Age (*M* [SD])	24.1 (5.6)
Body Mass Index (*M* [SD])	25.3 (6.2)
Gender	
Cisgender Boy/Man	82.6
Trans Boy/Man	7.6
Gender Expansive or Other	9.8
Race/Ethnicity	
Asian	10.7
Black	3.1
Latin American	4.1
Multi-Racial	13.0
Other	3.5
White	65.6
Sexual Orientation	
Heterosexual	48.2
Gay	19.5
Bisexual	13.8
Queer	8.1
Questioning or Other	10.5
Highest Completed Education	
High School Diploma or Less	44.1
College or Undergraduate Degree	39.8
Master’s Degree or Higher	16.1
Country	
Canada	58.3
United States	41.7
Eating Disorder or Muscle Dysmorphia Diagnosis	
None	78.2
Anorexia Nervosa/Atypical Anorexia Nervosa	3.9
Bulimia Nervosa	9.1
Binge-Eating Disorder	4.2
Muscle Dysmorphia	4.5
Intentions to Use Anabolic-Androgenic Steroids (*M* [SD])	8.3 (5.9)

*M* Mean,* SD* Standard deviation

**Table 2:** Associations between probable eating Disorders, probable muscle Dysmorphia, and intentions to use Anabolic-Androgenic steroids among never consumers

	B (95% CI)	*p*
None	Ref.	Ref.
Anorexia Nervosa/Atypical Anorexia Nervosa	**3.24 (1.05–5.43)**	**0.004**
Bulimia Nervosa	**3.27 (1.82–4.71)**	**< 0.001**
Binge-Eating Disorder	-1.31 (-3.24-0.63)	0.186
Muscle Dysmorphia	**3.16 (1.31–5.01)**	**0.001**

This table represents the abbreviated output of one linear regression analysis with probable eating disorder, probable muscle dysmorphia, or none as the independent variable and intentions to use anabolic-androgenic steroids as the dependent variable. Analysis adjusted for age, body mass index, gender, race/ethnicity, sexual orientation, highest completed education, and country.

**Boldface** indicates statistical significance at *p* < .05.

CI Confidence interval

**Fig. 1:**
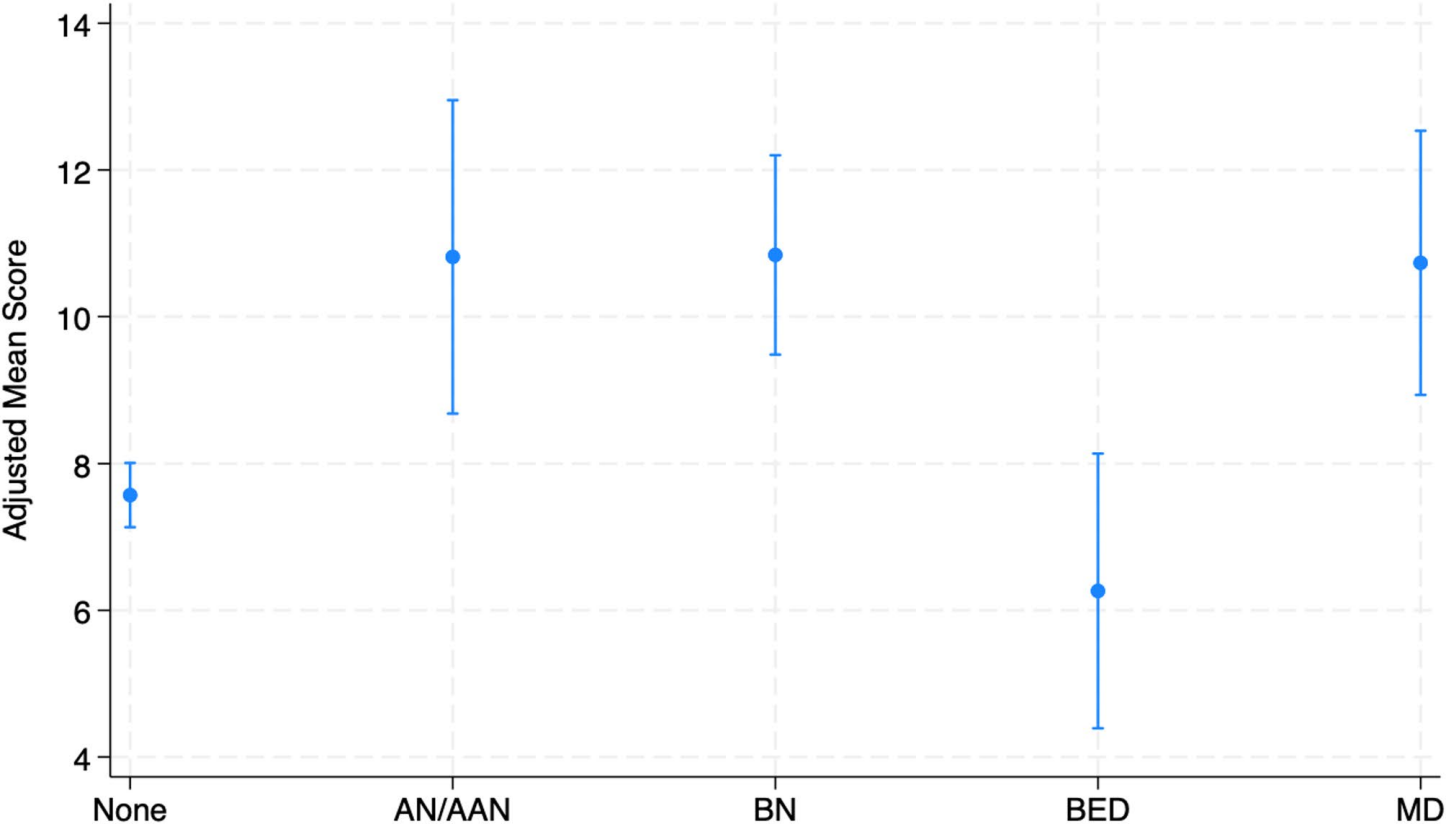
Group Differences in Adjusted Mean Intentions to Use Anabolic-Androgenic Steroids. Significant (*p* < .05) differences in mean scores determined via post-hoc Wald tests: AN/AAN vs. BN: *p* = .983; AN/AAN vs. BED: *p* = .001; AN/AAN vs. MD: *p* = .955; BN vs. BED: *p* < .001; BN vs. MD: *p* = .926; BED vs. MD: *p* = .001. Error bars equal standard deviation. *AN/AAN* anorexia nervosa/atypical anorexia nervosa, *BN* bulimia nervosa, *BED* binge-eating disorder, *MD* muscle dysmorphia

Group differences in adjusted mean intentions to use AAS scores are displayed in Fig. 1. Post-hoc Wald tests revealed significant differences in scores between probable AN/AAN and probable BED, probable BN and probable BED, and probable BED and probable MD.

## Discussion

This study aimed to investigate whether boys and men with a probable ED or probable MD have greater intentions to use AAS compared to those with neither condition and whether there are differences in AAS use intentions between those with probable EDs and probable MD. The findings confirmed the study hypotheses that boys and men with a probable ED, namely probable AN/AAN and probable BN, or probable MD, had significantly greater intentions to use AAS compared to those without these conditions. Additionally, the findings demonstrated significant differences in associations with intentions to use AAS between those with probable BED and all other conditions under study, with those with probable BED having significantly lower intentions to use AAS. There were no differences in intentions to use AAS between those with probable AN/AAN, probable BN, and probable MD. These are novel findings as they expand prior research that has predominantly focused on relatively small samples or participants from a particular group (e.g., students, bodybuilders). Additionally, the exploration of these associations using clinical criteria for EDs and MD, versus measurement of symptomatology on a continuum, provides additional support for the overlapping symptoms and behaviors of these conditions.

Boys and men experience significant sociocultural pressures from the media, family, and peers to adhere to the muscular body ideal [47]. Given these pressures, the overall greater intentions to use AAS among those with probable AN/AAN, probable BN, and probable MD, compared to those with none of these conditions, is likely driven by the significant body image preoccupation and body dissatisfaction that underpin these conditions [21, 22, 25, 66]. Conversely, there was no significant difference in intentions to use AAS among those with probable BED and those without, which is interesting given emerging evidence suggesting high body image concerns among this population [67]. For both those with probable AN/AAN and probable BN, intentions to use AAS may stem from a desire to increase leanness and muscularity [27], potentially functioning as a form of compensatory behavior aimed at achieving a more muscular physique to align with the sociocultural ideal [24]. Prior research has also reported a positive association between purging behaviors (e.g., self-induced vomiting) and AAS use [37], highlighting that these behaviors likely do not occur in isolation and may be indicative of an overall symptom pattern for these conditions.

Regarding those with probable MD, there is ample evidence that AAS use occurs among those with this condition [32–34], due to the primary focus of this condition being on drive for muscularity and muscle dissatisfaction. Indeed, given the effectiveness of AAS at increasing muscle mass [31], considering the use of these drugs is highly relevant among this cohort. Additionally, intentions to use AAS (i.e., talking to people about them, looking up information about them) among those with probable MD may be considered an obsessive and compulsive symptom, as these behaviors could alleviate thoughts focused on insufficient muscularity [68]. Taken together, these patterns highlight how intentions to use AAS may emerge from the underlying psychopathology of MD, while also intersecting with the broader spectrum of eating- and body-related disorders in boys and men.

Despite being classified differently [21], AN/AAN, BN, and MD among boys and men have overlapping features that likely underpin the lack of differences in intentions to use AAS found in this study. For example, boys and men with these conditions primarily experience body image concerns that focus on muscle dissatisfaction, frequently inspect their musculature, and engage in behaviors to increase their muscularity (e.g., excessive weight training, dietary changes) [22–26]. Indeed, there is research documenting an association between MD and ED symptoms [19]. Therefore, comparable intentions to use AAS may be representative of another behavioral similarity across these conditions. Such data may provide relevant evidence to support changes in future iterations of diagnostic criteria and the classification of these conditions [20, 40].

Critically, the analyses in this study adjusted for several sociodemographic identifiers that have previously been identified as important when considering EDs and MD. For example, the findings are independent of participants’ age, which is relevant given that the mean age of onset for an ED or MD is between 19 and 21 years [12, 13]. Additionally, sociocultural pressures and gendered norms and expectations may differ between adolescent boys and young adult men, which may reasonably be expected to change intentions to use AAS. Thus, the findings indicate that intentions to use AAS across probable AN/AAN, probable BN, and probable MD are similar across the ages in this study. The findings are also independent of gender, sexual orientation, and race/ethnicity, which prior research has documented that gender, sexual, and racial/ethnic minority boys and men may experience greater ED and MD symptoms compared to their cisgender, heterosexual, and white peers [69–72]. Therefore, the findings provide support for the notion that intentions to use AAS are similar across boys and men with probable AN/AAN, probable BN, and probable BED of diverse genders, sexual orientations, and race/ethnicities. However, there remains a significant need for health care professionals to be trained and prepared to assess and treat boys and men of diverse identities with these conditions, given the nuances of body ideals and gender norms and expectations. Finally, the findings are independent of country of residence, which is not surprising given that both Canada and the U.S. have similar regulations of AAS, as well as sociocultural environments that perpetuate specific body ideals.

The findings from this study should be contextualized by several limitations, which can be addressed in future research. The sample was recruited using non-probability sampling techniques, which introduces limitations in the generalizability of the findings. However, the sample was diverse across sociodemographic variables, and participants represented all 13 Canadian provinces and territories, as well as all 10 major zip code (i.e., geographic) regions of the United States. Nevertheless, investigating intentions to use AAS among nationally representative samples is warranted to inform public health prevention and interventions. A probable ED and MD were not assessed through clinical interviews in this study. However, the study employed an algorithm based on clinically defined criteria from the DSM-5 [15, 16]. This method improved reliability and allowed for a practical and cost-efficient method for large-scale studies. Future research is needed to extend these findings to clinical samples, including qualitative research that examines the mechanisms underpinning intentions to use AAS across those with EDs or MD. Similarly, the MDDI has not been formally validated among adolescents, which should be considered given the use of several items from this scale in the MD algorithm. The intentions to use AAS measure is not a validated measure and instead was based on prior research [53], which underscores the need for the development of a validated measure to assess intentions, attitudes, and risk of using AAS. Additionally, it is possible that there was an underreporting of intentions to use AAS due to social desirability bias (i.e., participants may not have wanted to disclose intentions to use AAS, given the undesirable nature of this behavior) and threat of disclosure (i.e., fears of possible risks or adverse effects from admitting intentions to use AAS) [73]. This underreporting may have been exacerbated by the fact that the sample was recruited online. Relatedly, the study was limited to assessing intentions to use AAS, rather than other appearance- and performance-enhancing drugs and substances (APEDS), such as selective androgen receptor modulators (SARMs), clenbuterol, stimulants, and dietary supplements (e.g., creatine, pre-workout). Future research should consider these additional APEDS, particularly given that they are commonly used alongside AAS [11, 74]. Finally, the study was cross-sectional in design, which limits the ability to determine the directionality between the primary variables under study. Future research should use longitudinal designs to investigate the directionality of the associations found in this study. Additionally, longitudinal research should investigate the processes that lead to the initiation of AAS use among individuals with EDs or MD, including statistical methods (e.g., structural equation modeling) that examine the mechanisms underlying these associations.

## Conclusion and implications

Intentions to use AAS were higher among boys and men with probable AN/AAN, probable BN, and probable MD compared to those with none of these conditions, and there were no differences in intentions to use AAS across these conditions. These findings suggest a need to develop targeted AAS prevention and intervention programs specifically for boys and men with AN/AAN, BN, and MD. Such programming may be grounded in the Health Belief Model (HBM) [75] and mirror the ATLAS Program [76], which underscores that knowledge about AAS, including its pros and cons of use and potential health effects, may be effective in deterring initiation [77]. The HBM has further utility in the design and execution of interventions related to changing AAS usage behaviors, where increased knowledge and cues to action can translate into safer behaviors, such as reduced AAS use [78, 79]. Healthcare professionals who engage with boys and men with an ED or MD should consider receiving education and training on AAS to gain the knowledge and confidence to assess and treat clients with intentions to use. As described by adapted models of the Tripartite Influence Model [47], integrated clinical treatment approaches may be needed to address the convergence of sociocultural pressures, masculine gender norms, body image concerns, and drive for muscularity that may influence intentions to use AAS among boys and men with an ED or MD. Finally, the findings from this study may provide further evidence that, while ED and MD are distinct conditions [21], they do overlap [19] and have similar symptomatology. Ultimately, these similarities may inform future diagnostic categorization and criteria, which would have significant implications, possibly leading to more equitable assessment, diagnosis, and treatment of EDs and MD among boys and men.

## Supplementary Information

Below is the link to the electronic supplementary material.

Supplementary Material 1.

## Data Availability

Data may be made available upon reasonable request.
