# Supplementary material for: Associations in intentions to use anabolic-androgenic steroids among non-consuming boys and men with probable eating disorders and muscle dysmorphia
Source: J Eat Disord. 2025 Nov 11;13:257. doi: 10.1186/s40337-025-01435-3 (PMC12607208; doi:10.1186/s40337-025-01435-3)
Supplement: Supplementary file 1 — Supplementary Material 1. [file 40337_2025_1435_MOESM1_ESM.docx]

| **Supplementary Table 1.**  Descriptive Statistics on the Intentions to Use Anabolic-Androgenic Steroids Measure among Non-Consumers (N = 1,515) | |
| --- | --- |
|  | Overall |
|  | % (*n*) |
| I plan to use anabolic-androgenic steroids in the future. (*M* [*SD*]) | 1.53 (1.12) |
| Strongly disagree (1) | 87.4 (1,121) |
| Disagree (2) | 3.2 (202) |
| Slightly disagree (3) | 5.6 (49) |
| Neither agree nor disagree (4) | 2.4 (85) |
| Slightly agree (5) | 2.4 (26) |
| Agree (6) | 0.6 (9) |
| Strongly agree (7) | 0.7 (11) |
| I have looked up information on types of anabolic-androgenic steroids and how to use them. (*M* [*SD*]) | 1.96 (1.74) |
| Strongly disagree (1) | 70.2 (1,062) |
| Disagree (2) | 8.5 (129) |
| Slightly disagree (3) | 2.6 (40) |
| Neither agree nor disagree (4) | 2.5 (37) |
| Slightly agree (5) | 8.4 (127) |
| Agree (6) | 5.3 (80) |
| Strongly agree (7) | 2.5 (37) |
| I have looked up information on how to obtain anabolic-androgenic steroids. (*M* [*SD*]) | 1.51 (1.31) |
| Strongly disagree (1) | 81.0 (1,225) |
| Disagree (2) | 8.1 (122) |
| Slightly disagree (3) | 2.1 (32) |
| Neither agree nor disagree (4) | 1.7 (26) |
| Slightly agree (5) | 3.2 (48) |
| Agree (6) | 2.4 (37) |
| Strongly agree (7) | 1.5 (23) |
| I've talked with people who use anabolic steroids about getting or using anabolic-androgenic steroids. (*M* [*SD*]) | 1.52 (1.30) |
| Strongly disagree (1) | 79.8 (1,204) |
| Disagree (2) | 9.7 (147) |
| Slightly disagree (3) | 1.8 (27) |
| Neither agree nor disagree (4) | 1.7 (26) |
| Slightly agree (5) | 2.8 (42) |
| Agree (6) | 3.0 (45) |
| Strongly agree (7) | 1.2 (18) |
| I've learned about getting and using anabolic-androgenic steroids. (*M* [*SD*]) | 1.80 (1.60) |
| Strongly disagree (1) | 73.6 (1,106) |
| Disagree (2) | 8.8 (133) |
| Slightly disagree (3) | 2.5 (37) |
| Neither agree nor disagree (4) | 2.5 (37 |
| Slightly agree (5) | 6.2 (94) |
| Agree (6) | 4.7 (71) |
| Strongly agree (7) | 1.7 (25) |
| *M* = Mean; *SD* = Standard deviation. |  |

| **Supplementary Table 2.**  Factor Loadings and Uniqueness Values for the Intentions to Use AAS Measure | | |
| --- | --- | --- |
| **Item** | **Loading** | **Uniqueness (u²)** |
| I plan to use anabolic-androgenic steroids in the future. | .68 | .54 |
| I have looked up information on types of anabolic-androgenic steroids and how to use them. | .83 | .31 |
| I have looked up information on how to obtain anabolic-androgenic steroids. | .85 | .28 |
| I've talked with people who use anabolic steroids about getting or using anabolic-androgenic steroids. | .71 | .50 |
| I've learned about getting and using anabolic-androgenic steroids. | .84 | .29 |
| Note. Extraction method = Principal factor analysis. One factor was specified. Loadings are standardized. | | |

| **Supplementary Table 3.**  Operationalization of DSM-5 Eating Disorders^1^ | | |
| --- | --- | --- |
| **Anorexia Nervosa** | | |
| Criteria^2^ | Operationalization^3^ | α^4^ |
| Restriction of energy intake relative to requirements, leading to a significantly low body weight in the context of age, sex, developmental trajectory, and physical health. Significantly low weight is defined as a weight that is less than minimally normal or, for children and adolescents, less than that minimally expected. | BMI of ≤ 18.5. BMI calculated based on self-reported weight and height (kg/m^2^) |  |
|  | **AND** |  |
| Intense fear of gaining weight or of becoming fat, or persistent behavior that interferes with weight gain, even though at a significantly low weight. | Any persistent extreme weight control behavior (≥ 4 in the past 28 days): |  |
|  | Fasting  EDE-Q Item 2: “Have you gone for long periods of time (8 waking hours or more) without eating anything at all in order to influence your shape or weight?” |  |
|  | Strict dieting  Researcher Developed Item: “Over the past 28 days, how many times have you followed a strict diet as a means of controlling your shape or weight?” |  |
|  | Detox  Researcher Developed Item: “Over the past 28 days, how many times have you followed a detox as a means of controlling your shape or weight?” |  |
|  | Self-induced vomiting  EDE-Q Item 16: “Over the past 28 days, how many times have you made yourself sick (vomit) as a means of controlling your shape or weight?” |  |
|  | Laxative use  EDE-Q Item 17: “Over the past 28 days, how many times have you taken laxatives as a means of controlling your shape or weight?” |  |
|  | Driven exercise  EDE-Q Item 18: “Over the past 28 days, how many times have you exercised in a “driven” or “compulsive” way as a means of controlling your weight, shape or amount of fat or to burn off calories?” |  |
|  | Misuse of insulin  Researcher Developed Item: “Over the past 28 days, how many times have you misused insulin as a means of controlling your shape or weight? “ |  |
|  | Diet pill use  Researcher Developed Item: “Over the past 28 days, how many times have you taken diuretics (water pills) or diet pills as a means of controlling your shape or weight?” |  |
|  | **OR** |  |
|  | Fear of weight gain  EDE-Q Item 10: “Have you had a definite fear that you might gain weight?” |  |
|  | **OR** |  |
|  | Felt fat  EDE-Q Item 11: “Have you felt fat?” |  |
|  | **AND** |  |
| Disturbance in the way in which one’s body weight or shape is experienced, undue influence of body weight or shape on self-evaluation, or persistent lack of recognition of the seriousness of the current low body weight. | Extreme weight/shape concerns over the past 4 weeks  ≥ 4 score on combined EDE-Q Weight and Shape Concerns scales. | 0.93 |
| **Bulimia Nervosa** | | |
| Criteria^2^ | Operationalization^3^ | α^4^ |
| Recurrent episodes of binge eating. An episode of binge eating is characterized by both of the following:  1. Eating, in a discrete period of time (e.g., within any 2-hour period), an amount of food that is definitely larger than what most individuals would eat in a similar period of time under similar circumstances.  2. A sense of lack of control over eating during the episode (e.g., a feeling that one cannot stop eating or control what or how much one is eating). | ≥ 4 objective binge eating episodes in past 4 weeks  EDE-Q Item 15: “Over the past 28 days, on how many DAYS have such episodes of overeating occurred (i.e., you have eaten an unusually large amount of food and have had a sense of loss of control at the time)?” |  |
|  | **AND** |  |
|  | Persistent extreme weight control behavior (≥ 4 in the past 28 days): |  |
|  | Fasting  EDE-Q Item 2: “Have you gone for long periods of time (8 waking hours or more) without eating anything at all in order to influence your shape or weight?” |  |
|  | Strict dieting  Researcher Developed Item: “Over the past 28 days, how many times have you followed a strict diet as a means of controlling your shape or weight?” |  |
|  | Detox  Researcher Developed Item: “Over the past 28 days, how many times have you followed a detox as a means of controlling your shape or weight?” |  |
|  | Self-induced vomiting  EDE-Q Item 16: “Over the past 28 days, how many times have you made yourself sick (vomit) as a means of controlling your shape or weight?” |  |
|  | Laxative use  EDE-Q Item 17: “Over the past 28 days, how many times have you taken laxatives as a means of controlling your shape or weight?” |  |
|  | Driven exercise  EDE-Q Item 18: “Over the past 28 days, how many times have you exercised in a “driven” or “compulsive” way as a means of controlling your weight, shape or amount of fat or to burn off calories?” |  |
|  | Misuse of insulin  Researcher Developed Item: “Over the past 28 days, how many times have you misused insulin as a means of controlling your shape or weight? “ |  |
|  | Diet pill use  Researcher Developed Item: “Over the past 28 days, how many times have you taken diuretics (water pills) or diet pills as a means of controlling your shape or weight?” |  |
|  | **AND** |  |
| Self-evaluation is unduly influenced by body shape and weight. | Extreme weight/shape concerns over the past 4 weeks  ≥ 4 score on combined EDE-Q Weight and Shape Concerns scales. | 0.93 |
|  | **AND** |  |
| The disturbance does not occur exclusively during episodes of anorexia nervosa. | Not meeting criteria for anorexia nervosa  See criteria outlined above. |  |
| **Binge-Eating Disorder** | | |
| Criteria^2^ | Operationalization^3^ | α^4^ |
| Recurrent episodes of binge eating. An episode of binge eating is characterized by both of the following:  1. Eating, in a discrete period of time (e.g., within any 2-hour period), an amount of food that is definitely larger than what most people would eat in a similar period of time under similar circumstances.  2. A sense of lack of control over eating during the episode (e.g., a feeling that one cannot stop eating or control what or how much one is eating). | ≥ 4 objective binge eating episodes in past 4 weeks  EDE-Q Item 15: “Over the past 28 days, on how many DAYS have such episodes of overeating occurred (i.e., you have eaten an unusually large amount of food and have had a sense of loss of control at the time)?” |  |
|  | **AND** |  |
| The binge-eating episodes are associated with three (or more) of the following:  1. Eating much more rapidly than normal.  2. Eating until feeling uncomfortably full.  3. Eating large amounts of food when not feeling physically hungry.  4. Eating alone because of feeling embarrassed by how much one is eating.  5. Feeling disgusted with oneself, depressed, or very guilty afterward. | Binge eating associated with ≥ 3 features:  Researcher Developed Item: “Over the past 28 days, during binge eating episodes, have you experienced any of the following?” |  |
|  | Rapid eating or eating quickly |  |
|  | Eating until you felt uncomfortably full |  |
|  | Eating despite not being hungry |  |
|  | Eating alone or in secret |  |
|  | Feeling disgusted, guilty, shameful, and/or depressed after binge eating |  |
|  | **AND** |  |
| Marked distress regarding binge eating is present. | Feeling disgusted, guilty, shameful, and/or depressed after binge eating |  |
|  | **AND** |  |
| The binge eating is not associated with the recurrent use of inappropriate compensatory behavior as in bulimia nervosa and does not occur exclusively during the course of bulimia nervosa or anorexia nervosa. Not meeting criteria for anorexia nervosa or bulimia nervosa | See criteria outlined above. |  |
| **Atypical Anorexia Nervosa** | | |
| Criteria^2^ | Operationalization^3^ | α^4^ |
| The individual’s weight is within or above the normal range. | Current BMI  BMI of > 18.5. BMI calculated based on self-reported weight and height (kg/m^2^) |  |
|  | **AND** |  |
| Significant weight loss has occurred. | Lost weight in the past 4 weeks  Researcher Developed Item: “Have you lost weight in the past 28 days?” |  |
|  | **AND** |  |
| Anorexia Nervosa Criterion B: Intense fear of gaining weight or of becoming fat, or persistent behavior that interferes with weight gain, even though at a significantly low weight. | Any persistent extreme weight control behavior (≥ 4 in the past 28 days): |  |
|  | Fasting  EDE-Q Item 2: “Have you gone for long periods of time (8 waking hours or more) without eating anything at all in order to influence your shape or weight?” |  |
|  | Strict dieting  Researcher Developed Item: “Over the past 28 days, how many times have you followed a strict diet as a means of controlling your shape or weight?” |  |
|  | Detox  Researcher Developed Item: “Over the past 28 days, how many times have you followed a detox as a means of controlling your shape or weight?” |  |
|  | Self-induced vomiting  EDE-Q Item 16: “Over the past 28 days, how many times have you made yourself sick (vomit) as a means of controlling your shape or weight?” |  |
|  | Laxative use  EDE-Q Item 17: “Over the past 28 days, how many times have you taken laxatives as a means of controlling your shape or weight?” |  |
|  | Driven exercise  EDE-Q Item 18: “Over the past 28 days, how many times have you exercised in a “driven” or “compulsive” way as a means of controlling your weight, shape or amount of fat or to burn off calories?” |  |
|  | Misuse of insulin  Researcher Developed Item: “Over the past 28 days, how many times have you misused insulin as a means of controlling your shape or weight? “ |  |
|  | Diet pill use  Researcher Developed Item: “Over the past 28 days, how many times have you taken diuretics (water pills) or diet pills as a means of controlling your shape or weight?” |  |
|  | **OR** |  |
|  | Fear of weight gain  EDE-Q Item 10: “Have you had a definite fear that you might gain weight?” |  |
|  | **OR** |  |
|  | Felt fat  EDE-Q Item 11: “Have you felt fat?” |  |
|  | **AND** |  |
| Anorexia Nervosa Criterion C: Disturbance in the way in which one’s body weight or shape is experienced, undue influence of body weight or shape on self-evaluation, or persistent lack of recognition of the seriousness of the current low body weight. | ≥ 4 score on combined EDE-Q Weight and Shape Concerns scales. | 0.93 |
|  | **AND** |  |
| Not meeting criteria for anorexia nervosa, bulimia nervosa, nor binge-eating disorder. | See criteria outlined above. |  |
| BMI = Body mass index; KG = Kilograms; M = Meters; EDE-Q = Eating Disorder Examination Questionnaire; OSFED = Other Specified Feeding and Eating Disorder  ^1^ Based on Mitchison, D., Mond, J., Bussey, K., Griffiths, S., Trompeter, N., Lonergan, A., Pike, K. M., Murray, S. B., & Hay, P. (2020). DSM-5 full syndrome, other specified, and unspecified eating disorders in Australian adolescents: Prevalence and clinical significance. *Psychological Medicine*, *50*(6), 981–990. <https://doi.org/10.1017/S0033291719000898>  ^2^ Criteria outlined in the Diagnostic and Statistical Manual of Mental Disorders, Fifth Edition: American Psychiatric Association, issuing body. (2022). *Diagnostic and statistical manual of mental disorders: DSM-5-TR* (5th edition, text revision.).  ^3^ Participants completed all items and measures within the Operationalization column.  ^4^ Internal consistency using Cronbach’s alphas of all full measures included in the operationalization among participants from The Study of Boys and Men (N = 1,553). | | |

| **Supplementary Table 4.**  Muscle dysmorphia diagnostic criteria^1^ and operationalization in the current study^2^ | | |
| --- | --- | --- |
| Criteria | Operationalization | α^5^ |
| A. The person has a preoccupation with the idea that one’s body is not sufficiently lean and muscular. Characteristic associated behaviors include long hours of lifting weights and excessive attention to diet | ≥ 1.5 SD above mean on the DFS subscale of the MDDI^3^  To enhance the validity of Mitchison et al.'s (2021) original operationalization, the current study defined a participant's preoccupation with muscularity as having a score of ≥ 1.5 standard deviations above the mean on the Drive for Size subscale of the Muscle Dysmorphic Disorder Inventory (Hildebrandt et al., 2004). | MDDI DFS subscale: 0.86 |
| B. The preoccupation causes clinically significant distress or impairment in social, occupational, or other important areas of functioning, as demonstrated by at least two of the following four criteria: | At least 2 of the following: |  |
| (a) The individual frequently gives up important social, occupational, or recreational activities because of a compulsive need to maintain his or her workout and diet schedule; | A score of ≥4 (indicating often to always) on one or more of the following DMS/MDDI items:  1. “I think that my weight-training schedule interferes with other aspects of my life” 2. “I pass up social activities with friends because of my workout schedule” 3. “I pass up chances to meet new people because of my workout schedule” |  |
| (b) The individual avoids situations where his or her body is exposed to others, or endures such situations only with marked distress or intense anxiety; | A score of ≥ 4 (indicating moderately to markedly) on the EDE-Q item:  “Over the past 4 weeks (28 days), how uncomfortable have you felt about others seeing your shape or figure (for example, in communal changing rooms, when swimming, or wearing tight clothes)?” |  |
| (c) The preoccupation about the inadequacy of body size or musculature causes clinically significant distress or impairment in social, occupational, or other important areas of functioning; | PedsQL physical or psychosocial score > 1 SD below the sample mean (indicating significant impairment)  OR  K10 total score ≥ 15 (indicating mild to severe distress) | PedsQL Adolescent: 0.86  PedsQL Young Adult: 0.88  K10: 0.92 |
| (d) The individual continues to work out, diet, or use ergogenic (performance enhancing) substances despite knowledge of adverse physical or psychological consequences | Score ≥ 4 (indicating often to always) on the DMS item: “I lift weights to build up muscle”  OR  Score ≥ 12 days on the researcher-developed item: “In the past 28 days, how many days have you been on a very high-protein diet as a means of controlling your shape or weight?”  OR  Score ≥ 1 on the researcher-developed item: “In the past 28 days, how many times have you taken anabolic steroids as a means of increasing your muscularity?” |  |
| C. The primary focus of the preoccupation and behaviors is on being too small or inadequately muscular, as distinguished from fear of being fat, as in anorexia nervosa, or a primary preoccupation only with other aspects of appearance, as in other forms of BDD | Score ≥ 4 (indicating often to always) on the DMS item: “I wish that I were more muscular”  AND  Exclusion if meeting criteria for anorexia nervosa, bulimia nervosa, atypical anorexia nervosa, or subthreshold bulimia nervosa^4^ |  |
| SD = Standard deviation; DFS = Drive for Size; MDDI = Muscle Dysmorphic Disorder Inventory; DMS = Drive for Muscularity Scale; EDE-Q = Eating Disorder Examination Questionnaire; PedsQL = Pediatric Quality of Life Inventory; K10 = Kessler 10; BDD = Body dysmorphic disorder  ^1^ Based on Pope, H. G., Gruber, A. J., Choi, P., Olivardia, R., & Phillips, K. A. (1997). Muscle dysmorphia: An underrecognized form of body dysmorphic disorder. *Psychosomatics*, *38*(6), 548–557. <https://doi.org/10.1016/S0033-3182(97)71400-2>  ^2^ Adapted from Mitchison, D., Mond, J., Griffiths, S., Hay, P., Nagata, J. M., Bussey, K., … Murray, S. B. (2022). Prevalence of muscle dysmorphia in adolescents: findings from the EveryBODY study. *Psychological Medicine*, *52*(14), 3142–3149. doi:10.1017/S0033291720005206  ^3^ Only minor change to Mitchison et al.’s operationalization.  ^4^ For eating disorder criteria, see Mitchison, D., Mond, J., Bussey, K., Griffiths, S., Trompeter, N., Lonergan, A., … Hay, P. (2020). DSM-5 full syndrome, other specified, and unspecified eating disorders in Australian adolescents: prevalence and clinical significance. *Psychological Medicine*, *50*(6), 981–990. doi:10.1017/S0033291719000898  ^5^ Internal consistency using Cronbach’s alphas of all full measures included in the operationalization among participants from The Study of Boys and Men (N = 1,553). | | |

| **Supplementary Table 5.**  Demographic Survey Questions | | | | |
| --- | --- | --- | --- | --- |
| Variable | Question | Original Survey Response Options | Recoded Categories | |
| Age | What is your current age? | Range: 15-35 | N/A | |
| Sex at birth | What sex were you assigned at birth on your original birth certificate? | Male Female Intersex Prefer not to say | 0=Female 1=Male  Note: There were no observations for “intersex” and “prefer not to say”. | |
| Gender identity | What is your current gender identity? Select all that apply. | Boy/man Girl/woman Trans male/Trans man Trans female/Trans woman Genderqueer/Gender non-conforming Gender non-binary  Self-identify (please specify)  Prefer not to say | 0=Boy/Man 1=Trans Man 2=Gender Expansive & Other  Note: “Prefer not to say” was coded as missing. | |
| Race/ethnicity | In our society, people are often described by their race/ethnicity or racial/ethnic background.   Which racial/ethnic category(ies) best describes you? Check all that apply. | Black (ex. African, African Canadian/American, Afro-Caribbean descent) East Asian (ex. Chinese, Japanese, Korean, Taiwanese descent) Indigenous (ex. First Nations, Inuk/Inuit, Métis descent) Latin American (ex. Hispanic or Latin American descent) Middle Eastern (ex. Arab, Persian, West Asian descent (e.g., Afghan, Egyptian, Iranian, Kurdish, Lebanese, Turkish)) South Asian (ex. South Asian descent (e.g., Bangladeshi, Indian, Indo-Caribbean, Pakistani, Sri Lankan)) Southeast Asian (ex. Cambodian, Filipino, Indonesian, Thai, Vietnamese, or other Southeast Asian descent) White (European descent [e.g., British, French, Italian, Portuguese, Ukrainian, Russian]))  Another race category (please specify) Do not know Prefer not to answer | 0=White 1=Black 2=Asian (East Asian, South Asian, Southeast Asian) 3=Latin American 4=Other (Middle Eastern, Indigenous, Other) 5=Multi-Racial  Note: “Prefer not to answer” and “Don’t know” were coded as missing. | |
| Sexual orientation | What is your current sexual orientation? Select all that apply. | Asexual Bisexual Gay/Lesbian Heterosexual ("straight") Pansexual Queer Questioning Other (please specify) Prefer not to say | 0=Heterosexual 1=Gay/Lesbian 2=Bisexual  3=Queer 4=Questioning, Other, Asexual, Pansexual, Other   Note: “Prefer Not to Say” were coded as missing. | |
| Highest completed education | What is the highest level of formal education that you have completed? | 8th Grade or lower 9th Grade 10th Grade 11th Grade 12th Grade/high school diploma or equivalent College diploma or trade school certificate University degree (e.g., BS, BA) Master's degree (e.g., MSc, MSW, MPH) Doctoral degree (e.g., PhD, MD, JD) Other (please specify) | 0=HS or less 1=College or undergrad degree 2=Master’s degree or higher  Note: “Other” responses were recategorized into their respective group. | |
| Location | What is your postal code or zip code?  Please use the following formats: "M5S 1V4" (postal code); "10001" (zip code) | Open response | 0=Canada  1=United States |  |

| **Supplementary Table 6.**  Descriptive Characteristics of a Sample of Boys and Men from Canada and the United States Who Never Used Steroids by Age and Country | | | | |
| --- | --- | --- | --- | --- |
|  | < 18 Years  (*n =* 190) | ≥ 18 Years  (*n =* 1,321) | Canada  (*n =* 769) | United States  (*n =* 549) |
|  | % | % | % | % |
| Age (*M* [SD]) | 16.1 (0.8) | 25.2 (5.0) | 25.0 (5.3) | 23.3 (5.6) |
| Body Mass Index (*M* [SD]) | 23.2 (5.7) | 25.6 (6.2) | 25.3 (6.1) | 25.5 (6.4) |
| Gender |  |  |  |  |
| Cisgender Boy/Man | 79.3 | 83.0 | 88.3 | 83.4 |
| Trans Boy/Man | 13.3 | 6.8 | 5.8 | 10.4 |
| Gender Expansive or Other | 7.4 | 10.2 | 5.8 | 6.2 |
| Race/Ethnicity |  |  |  |  |
| Asian | 7.3 | 11.1 | 15.3 | 5.5 |
| Black | 4.0 | 3.0 | 2.1 | 5.0 |
| Latin American | 5.7 | 3.9 | 2.6 | 6.6 |
| Multi-Racial | 13.0 | 13.0 | 11.1 | 15.8 |
| Other | 4.5 | 3.4 | 4.9 | 1.8 |
| White | 65.5 | 65.6 | 64.0 | 65.3 |
| Sexual Orientation |  |  |  |  |
| Heterosexual | 52.3 | 47.6 | 49.1 | 43.9 |
| Gay | 14.6 | 20.2 | 19.8 | 20.9 |
| Bisexual | 19.7 | 12.9 | 12.8 | 15.6 |
| Queer | 2.2 | 8.9 | 8.7 | 7.5 |
| Questioning or Other | 11.2 | 10.4 | 9.6 | 12.1 |
| Highest Completed Education |  |  |  |  |
| High School Diploma or Less | 100.0 | 36.4 | 36.1 | 51.8 |
| College or Undergraduate Degree | - | 45.3 | 46.2 | 33.2 |
| Master’s Degree or Higher | - | 18.3 | 17.7 | 15.0 |
| Country |  |  |  |  |
| Canada | 37.3 | 60.9 | - | - |
| United States | 62.7 | 39.1 | - | - |
| Eating Disorder or Muscle Dysmorphia Diagnosis |  |  |  |  |
| None | 75.2 | 78.6 | 78.3 | 79.6 |
| Anorexia Nervosa/Atypical Anorexia Nervosa | 3.7 | 4.0 | 3.7 | 4.0 |
| Bulimia Nervosa | 8.3 | 9.3 | 9.2 | 7.8 |
| Binge-Eating Disorder | 5.5 | 4.0 | 4.4 | 4.0 |
| Muscle Dysmorphia | 7.3 | 4.2 | 4.4 | 4.6 |
| Intentions to Use Anabolic-Androgenic Steroids (*M* [SD]) | 9.5 (6.5) | 8.2 (5.7) | 8.8 (6.3) | 7.8 (5.2) |
| K10 Score (*M* [SD]) | 25.7 (9.2) | 24.7 (8.8) | 24.9 (8.9) | 24.2 (8.9) |
| *M* = Mean; SD = Standard deviation; K10 = Kessler Psychological Distress Scale | | | | |
